# Supplementary material for: Are carbon emissions trading and green financial instruments synergistic? -Comprehensive quantitative research based on content analysis
Source: PLoS One. 2024 Mar 7;19(3):e0298601. doi: 10.1371/journal.pone.0298601 (PMC10919601; doi:10.1371/journal.pone.0298601)
Supplement: S1 Data — (ZIP) [file pone.0298601.s001.zip › Data/地级市Z_F工作报告.zip!/╔╜╢1⁄2/┴┘╥╩╩╨/└┤╫╘╣1⁄2╓┌║┼í╛┬φ┐╦╩2╛▌═°í┐_2019.docx]

各位代表：

现在，我代表市人民政府向大会报告工作，请予审议，并请市政协委员和其他列席会议的同志提出意见。

一、2018年工作回顾

过去一年，在市委坚强领导下，我们坚持以习近平新时代中国特色社会主义思想为指导，认真贯彻中央和省委、省政府决策部署，解放思想求突破、凝心聚力快发展，统筹做好稳增长、促改革、调结构、惠民生、防风险各项工作，经济社会迈上高质量发展新台阶，大美新临沂建设展现更加亮丽的风采！

——经济发展稳中有进、进中向优。面对年初主要指标大幅下滑的严峻局面，加强分析研判和运行调度，强化精准施策，千方百计破解发展瓶颈制约。大力推进“双招双引”，新引建项目970个，到位市外资金850亿元；引进“两院”院士、国家“千人计划”专家等高层次人才209名，带资金、带技术、带项目的创新创业人才1100人。深化政银企合作，举办第五届资本交易大会，金融机构本外币贷款余额新增614亿元，增长13.7%。用好国家土地二级市场试点政策，盘活闲置土地8879亩、低效用地4303亩，实现供地5.5万亩。落实鼓励非公有制经济发展的政策，为企业减税降费108亿元、出口退税37.6亿元。实行领导干部帮包企业制度，69名市直机关县级干部联系105个企业项目，帮助解决实际困难和问题352个。目前，全市经济“稳”的基础更加牢固，“进”的势头更加明显，“优”的成效更加突出。预计全年完成生产总值4750亿元，增长7.5%；一般公共预算收入311.8亿元、增长9.3%，税收占比84.7%、居全省第1位；三次产业比例调整到7.7：44.1：48.2；固定资产投资、社会消费品零售总额、出口额分别增长7.8%、8.6%和8.3%；居民消费价格涨幅控制在2.6%以内。

——新旧动能转换全面起势。坚持规划引领，实行一个主导产业、一名市级领导、一个责任部门、一套工作班子、一个实施方案的“五个一”推进机制，完善人才、土地、金融、财税、审批服务、项目招引、政务环境和督导考核八条服务保障线，落实产业培育、项目引建和创新驱动等关键措施，加快存量变革、增量崛起。发展路径更加清晰。在全省率先获批新旧动能转换重大工程总体规划，明确“一城引领、两廊带动、三园集聚、全域协同”总体布局。对重点产业逐一制定专项规划，明确了主攻方向。坚决淘汰落后过剩产能。清理取缔“散乱污”企业3318家，淘汰钢铁产能285万吨、化工产能286万吨、非煤矿山产能600万吨，压减煤炭消费总量35万吨。改造提升传统产业。实施技改项目1176个、投资额增长18.5%。工程机械、木业、精品旅游、商贸物流4个产业集群，列入全省首批现代优势产业集群。营业收入过百亿元企业达到7家，5个品牌入选中国品牌价值分类百强榜。积极培育新兴产业。新增高新技术企业99家，信息技术、软件、康养等“四新”经济增加值增速均在15%以上。临港1400万吨高端不锈钢和特钢制造产业集群，纳入全省先进钢铁制造产业基地发展规划。电子元器件及其功能材料，成为国家创新型产业集群试点。创新驱动力增强。研发投入占生产总值比重达到2.5%，万人拥有有效发明专利2.82件，获省级及以上科技奖励11项。新增科技创新平台280个，中科（临沂）创新园正式开园，软磁复合材料制造业创新中心通过省级验收，中印科技国际创新园入选全省大数据产业集聚区。

——三大攻坚战取得积极进展。突出强弱项、补短板、固底板，筑牢发展底线。全力防范和化解重大风险。坚决守住金融风险底线，对贷款5亿元以上企业强化监测预警，对恶意逃废银行债务、非法融资、金融诈骗等行为依法严厉打击；对出险企业，加大“拆圈断链”、产权重组力度，强化综合施策，妥善处置了常林集团、江泉集团等融资风险问题。从严从实抓好安全生产工作，坚持“一个重点一个重点治理、一个行业一个行业整顿”，深入开展打非治违行动，安全生产形势有所好转。加强社会治安综合治理，扎实开展扫黑除恶专项斗争，打掉涉黑社会性质组织9个、恶势力犯罪团伙73个，查处涉黑涉恶“保护伞”47人。多措并举，妥善化解信访积案1411件。上合组织青岛峰会等重大活动期间，安保维稳实现“六不双零”目标。全力推进精准脱贫。聚焦“两不愁、三保障”，加大特困群众保障性兜底力度，新建产业扶贫项目340个、覆盖9.95万人，为28万人购买扶贫特惠保险，现行标准下建档立卡贫困人口、贫困村全部摘帽，扶贫工作基本完成三年任务，获得我省唯一的全国组织创新奖。全力推进污染防治。实施“四减四增”三年行动，抓好中央和省级环保督察反馈问题整改，空气质量同比改善7.9%，水体质量13个国省控断面考核指标均值全部达标，万元GDP能耗完成全年目标任务。新增造林面积15.8万亩，森林覆盖率达到22.8%，蒙阴县入选全国“绿水青山就是金山银山”实践创新基地。

——城乡面貌持续改善。实施新型城镇化战略和乡村振兴战略，加强城乡一体化规划、建设和管理。全市户籍人口城镇化率达到48.3%，顺利通过国家卫生城市、森林城市、园林城市复审，获得山东省适宜人居环境奖。城市功能进一步增强。同步推进老城改造和新城建设，新一轮城市发展战略研究基本完成，主城区控规实现全覆盖。北城二期、火车站、高铁、西部新城、相公等片区开发加快，完成征迁面积630万平方米。新增集中供热和清洁取暖面积920万平方米，新建海绵城市19平方公里、污水处理厂6座，集中供热、管道燃气、公共供水普及率分别达到71%、81%和84%。强力治理违章建设，拆除违建面积3700万平方米。重大基础设施推进顺利。鲁南高铁临沂段完成工程量的80%，京沪高速改扩建和岚罗、新台高速公路建设完成节点任务，新西外环、中环北线、罗程路改造、南外环大修等道路工程竣工通车。临枣1000千伏特高压开工建设，临潍1000千伏特高压全线架通。岸堤水库除险加固等72项重点水利工程全面开工，第二水源地启动实施。乡村振兴开局良好。推动主要农产品稳产提质，粮食、蔬菜、肉蛋奶产量分别达到409万吨、1418万吨、190万吨，新认证“三品一标”53个。推进30个田园综合体建设，示范带动农村“新六产”发展，“朱家林”经验列入全国首个田园综合体地方标准体系。加大新型农业经营主体培育力度，培训农民20万人次，新创建省级以上农业产业化龙头企业19家。扎实开展乡风文明和美丽乡村建设，改造农村危房5678户，改造旱厕13.7万户，新改建“四好农村路”4974公里。

——改革开放纵深推进。注重向改革要动力、以开放增活力，掀起了新一轮改革开放热潮。思想观念得到更新。组织党政代表团赴南方四市考察，实施对标赶超计划，制定差异化考核办法，推动了全市各级各部门思想大解放，增强了改革创新、换道超车的紧迫感。机制障碍加快破解。推进以“一次办好”为重点的“放管服”改革，市县行政权力事项分别压减34%和27%，涉企营业执照“45证合1”，建设工程“多图联审”模式在全省率先推行，行政审批“3124”目标基本实现，新登记各类市场主体增长29%。国有集体企业规范化公司制改制基本完成，91家市属经营性国有资产实现统一监管。在市属开发区推行大部门、全员聘任和绩效工资制，激发内生动力，省级以上开发区税收增长12%。扎实推进农村集体产权制度改革，98%的村完成改革任务。深入推进财税、教育、科技、卫生等领域改革，社会事业发展更具生机。对外开放持续扩大。临沂商城国际化、电商化水平提高，“市场采购”贸易方式出口额、物流总额、网络交易额分别达到73.4亿元、7300亿元和2200亿元，增长17%、8.8%和27%。涉外服务体系日趋完善，综合保税区进出口总额突破18亿美元、增长126%。“齐鲁号”欧亚班列、5条国际航线和2条全货机航线成功开行，临沂机场年旅客吞吐量突破200万人次。

——民生保障更加有力。实施十大领域重点惠民工程，努力增加基本公共服务供给。各级财政民生支出516.7亿元，占一般公共预算支出80.8%；城镇和农村居民人均可支配收入分别达到35727元、13638元，增长7.4%和8.1%。就业创业更有活力。新增城镇就业12.4万人，转移农村劳动力就业10.5万人，城镇登记失业率控制在2.27%以内。社会保障更有温度。企业退休人员养老金连续14年提高，19.3万名职工享受失业稳岗补贴，9.6万名残疾人得到康复救助，我市在中国城市慈善指数排名中列第13位、居全省首位。住房保障更有力度。房地产开发投资和商品房销售面积分别增长24.8%、7.1%，改造棚户区4.85万套，整治老旧小区285.6万平方米，政府投资公租房分配率100%。教育发展更有质量。新建、改扩建中小学校109所、幼儿园111处，全面改薄任务基本完成。鲁南技师学院成为国家职业教育示范校，临沂大学成为硕士学位授予单位。医疗服务更有效率。建设医联体、医共体127个，基层医疗卫生机构标准化建设达标率93%。在全省率先取消医用耗材加成，减轻群众看病负担2亿元。文体旅事业更有品位。行政村基本建立综合文化服务中心，我市被评为第三届文化强省建设先进市。成功举办世界皮划艇冠军挑战赛等赛事，参加省全运会获得金牌总数实现历史性突破。全域旅游快速发展，旅游消费额增长13%，中俄红色旅游合作交流系列活动有效开展。公共安全更有保障。学校食堂、旅游景区餐饮单位监管和食品药品安全审稽实现全覆盖，食安山东品牌达到774个。人民防空、应急管理、地震气象工作全面加强，安全保障水平进一步提高。

——政务生态环境不断优化。认真履行政府系统管党治党主体责任，坚持从严治政，政府工作人员忠诚干净担当的行动自觉进一步增强。严格依法行政，自觉接受各方面监督，人大建议和政协提案办结率100%。深化政务公开，完善群众诉求解决机制，市政务服务热线群众诉求事项办结率99%以上。协调发展各项事业，国防动员、妇女儿童、民族、宗教、史志、档案、对台、侨务和对口支援等工作都取得新成绩。深入开展大学习、大调研、大改进，持续加强纪律作风整顿，全市各级政府及部门的行政效能、工作质量进一步提升。我市新争取到首批全国黑臭水体治理示范城市、建筑垃圾治理试点城市、供应链创新与应用试点城市、校园足球改革实验区、全省“一带一路”建设综合试验区等85项省级以上试点，新获得全国商贸服务型国家物流枢纽承载城市、全国平安农机示范市等一批“国字号”荣誉。这些试点和荣誉，必将提升我市知名度、美誉度，提振广大干部群众争先创优的精气神，激励全市上下夺取一个又一个新胜利！

各位代表，发展伴随艰辛，成绩来之不易。这些成绩的取得，得益于党中央、国务院的亲切关怀，得益于省委、省政府和市委的正确领导，得益于市人大和市政协的监督帮助，得益于企业家们的开拓进取、创新创业，得益于全市人民的共同奋斗、无私奉献。在此，我代表市人民政府，向全市广大干部群众，向人大代表、政协委员、各民主党派、工商联、人民团体和离退休老同志，向驻临人民解放军、武警官兵和中央、省属单位，向关心支持临沂发展的社会各界朋友，表示衷心感谢和崇高敬意!

在肯定成绩的同时，我们也清醒认识到，我市发展正处在深度调整期、瓶颈突破期、动能转换胶着期，多种矛盾交织，发展不平衡不充分的问题仍然突出。主要表现在：综合实力不强，经济面临下行压力，内需增长有所放缓，外需拉动乏力，部分企业生产经营困难；发展质量效益不高，产业结构偏重，用能结构偏煤，创新能力薄弱的问题尚未根本性改变；各类风险隐患不断凸现，资源环境约束趋紧，污染物排放总量居高不下，安全生产形势依然严峻；民生领域历史欠账较多，群众对住房、出行、供暖、供水等公共服务还有一些不满意的地方；政府职能转变和制度创新力度不大，工作人员担当精神、专业素养、开放意识有待提升，营商环境离企业和群众期望还有差距；落实全面从严治党责任存在薄弱环节，党员干部违法违纪现象和腐败问题时有发生，等等。对上述问题，我们一定以更坚定的决心、更扎实的举措、更努力的工作，认真加以解决。

二、2019年工作安排

今年是新中国成立70周年，是全面建成小康社会的关键之年，也是加快新旧动能转换、实现高质量发展的重要一年。做好今年工作，我们必须深刻领会国家重要战略机遇期的内涵，抓住用好党中央、国务院深化改革开放的机遇，抓住用好省委、省政府强力推进新旧动能转换重大工程的机遇，抓住用好新技术革命、绿色发展、市场需求持续扩大、补齐民生短板等一系列机遇。只要我们深刻认识机遇，牢牢把握机遇，切实用好机遇，积极作为、砥砺奋进，就一定能够战胜各种困难和挑战，实现全年各项工作新突破！

今年政府工作的总体要求是：高举中国特色社会主义伟大旗帜，深入贯彻落实党的十九大精神和习近平总书记视察山东重要讲话、重要指示批示精神，牢牢把握“两个走在前列、一个全面开创”的目标定位，紧紧抓住用好重要战略机遇期，以高质量发展为根本要求，以弘扬沂蒙精神为动力，以“改革推进和工作落实年”为载体，突出抓好动能转换、扩大开放、城市建设、乡村振兴、深化改革、民生保障“六大重点”，奋力谱写新时代大美新临沂建设新篇章。

今年全市经济社会发展主要预期目标为：生产总值增长7.5%左右，一般公共预算收入增长7%，固定资产投资、社会消费品零售总额分别增长7%和8%左右，进出口保持稳定发展，城镇居民人均可支配收入增长7.5%左右，农村居民人均可支配收入增长快于城镇居民，全面完成省政府下达的约束性指标。

今年政府工作，突出抓好六个方面：

（一）强力推进新旧动能转换，加快实现高质量发展新突破。坚定不移推动高质量发展，把实施新旧动能转换重大工程作为引领高质量发展的总抓手。扎实做好我市第四次全国经济普查现场登记工作，构建新旧动能转换评价指标体系，为动能转换提供科学依据。正确处理稳与进、好与快、新与旧的关系，深化供给侧结构性改革，做好“压、转、育”三篇文章，推进先进制造业与现代服务业深度融合，着力做大产业、做强企业、做优品牌，推动经济发展行稳致远。一是壮大优势产业。以园区化、集群化、高端化为主攻方向，紧密对接省“十强”产业，壮大地方特色产业，发展未来潜力产业，抓好建链延链补链强链。重点发展先进装备制造、现代高效农业及食品加工、新材料、建筑建材、医养健康、文化旅游、现代商贸物流、高端化工、信息通讯技术、现代金融十大优势产业。实施“十优产业倍增”计划，今年全市实现产值过千亿级产业6个，经过3~5年努力，力争实现“十优产业”总产值（增加值）翻一番，成为在全省、全国具有一定影响力的产业集群。二是培植领军企业。实施“名企倍增”计划，选择符合高质量发展要求的部分龙头企业、潜力企业予以积极扶持，用3~5年时间实现产值和效益倍增。实施企业家培育计划，引导他们树立战略眼光，完善法人治理结构，心无旁骛攻主业，争当行业“单项冠军”。积极推进军民融合深度发展，发扬爱党爱军光荣传统，利用红色资源优势，找准军民融合结合点，拓宽“军转民”“民参军”渠道，推动军民融合红色教育、军民融合后勤保障社会化服务和军民融合产业合作取得新成效。三是打造知名品牌。扎实推进国家标准化综合改革试点，抓住质量、商标、专利“三个重点”，提升传统品牌、培植新兴品牌、打击假冒品牌。以高标准引领高质量，争取主导或参与制定国际、国家和行业标准40项以上。四是强化创新支撑。实施创新驱动发展战略，打造“政产学研金服用”创新共同体。推进临沂应用科学城二期、中科院计算技术研究院临沂分院建设，鼓励企业与高校、科研院所合作建设重点实验室、研究中心、技术中心等创新平台，协同攻克一批“卡脖子”技术难题，转化一批科技创新成果。力争每个优势产业至少建立1家市级以上研发中心，全市研发投入占生产总值比重提高到2.65%；新增省级以上科技创新平台50家，孵化面积达到190万平方米；高新技术产业产值占规模以上工业产值比重提高1个百分点。五是坚决淘汰落后过剩产能。严把行业准入门槛，倒逼落后过剩产能和“僵尸企业”市场出清，为“四新”经济发展腾出空间。力争淘汰铸造产能25万吨、化工产能300万吨、板材产能210万立方米。六是大力培育新的经济增长点。集中力量建设高端不锈钢与特钢生产加工基地和研发中心、交易中心，争取用3~5年时间产值突破3000亿元，使我市不锈钢产业成为国内领先、全球知名的产业集群。加快建设“沂蒙云谷”，积极推进郯城电子信息产业园建设，实施“优势制造业+人工智能”行动，着力推进互联网、云计算、大数据、人工智能与制造业深度融合，使“四新”经济成为我市高质量发展的新动能。发挥国家林产工业科技示范园载体优势，发展全屋定制、智慧精装等新业态，提升研发设计、展示交易服务水平，打造全国重要的绿色新型人造板产销基地。

（二）用好两个市场两种资源，加快实现对外开放新突破。坚定不移将改革开放进行到底，把发展开放型经济作为高质量发展的重要引擎。坚持进口和出口并重、招商引资和招才引智并行、利用外资和对外投资并举，建设内外贸融合发展示范区，努力把开放的门开得更大。提升商城“大市场”。加强政府引导，科学规划布局，促进传统市场升级换代、集约发展。完善临沂“商谷”基础设施配套，抓好进口商品城等项目建设，打造现代商城核心区。推动线上线下融合，推广新明辉、兰华集团等“触电”经验，培育垂直电商和跨境电商，力争2~3年建成区域性网货集散中心。发展现代物流，启动物流市场搬迁，在京沪高速两侧和济铁物流园规划建设一批“统仓统配”现代物流园区，布局一批特色地产品加工园区，增强商城发展支撑力。继续办好商博会、资本交易大会、秋季五金展、木博会等品牌展会，建设区域性会展中心城市。构建招商“大格局”。盯紧日韩、欧盟、港台等重点国家和地区，深度融入共建“一带一路”，绘制招商地图，建立招商项目库。压实责任，坚持重点项目定期调度制度，推动党政领导干部和职能部门带头招商。建立市场化招商队伍，聘请“招商顾问”，完善精准招商和项目落地机制，提高招引成功率。强化产业链招商，推动上下游关联企业集聚成群。抓好园区招商，鼓励发展“飞地经济”，吸引更多企业和项目“安营扎寨”。深入落实人才新政22条，继续实施人才集聚培育“五大工程”“六项计划”，努力留住本土人才，吸引更多外来人才。开辟协作“大通道”。主动融入淮海经济区、对接沿海城市群、建设临日都市区，在道路交通、产业布局、要素配置、政策争取等方面加强协调联动。扩大国际合作，支持有条件的骨干企业走出去开拓市场。健全跟踪、引进、合作、拓展的工作机制，推动“十优产业”与全国全球先进技术、高端产品、资本、人才、管理进行嫁接。完善涉外服务体系，用好“市场采购”贸易方式试点政策，提升综保区功能，健全国际营销网络，开行更多的国际货运班列，争取航空口岸正式开放，推动货畅其流。

（三）高质量推进城市建设，加快实现城市品质新突破。按照一流标准，打造宜居宜业宜游、现代化生态化特色化区域中心城市，进一步发挥主城带动作用。年内户籍人口城镇化率提高到49%。坚持高水平规划。强化“多规合一”，做到一张蓝图绘到底。启动新一轮城市总体规划编制，向北加快北城三期开发建设，向东规划国际生态城，向南加大罗庄片区旧城改造力度，向西将临沂商谷、高新区拓展至新西外环，进一步拉开城市框架。加强沭河两岸生态保护和开发，科学制定总体规划和控制性详细规划，有序推进中心城区由“一河为轴”逐步迈向“两河时代”。实行片区化开发。强化系统思维，统筹生产生活生态，加快高铁、奥体、相公、商谷、火车站、枣园等重点片区建设步伐。突出“一城绿色半城河”水城特色，联动开发“水、岸、滩”，一体打造“堤、路、景”，把生态自然美融入城市。加大配套设施补短板力度。实施281个城建重点项目，加快完善城市功能。新增供热能力260万平方米，改造供水管网80.3公里、燃气管网93.9公里，建设电力方涵6.4公里，建成临枣特高压交流输电工程。发展立体大交通。打造综合交通枢纽城市，促进对外快捷畅通、市域互联互通。推进鲁南高铁建设，确保年底建成通车，圆老区人民的高铁梦。继续抓好京沪高速改扩建、岚罗高速、新台高速、鲁南高铁临沂站、临沂机场改扩建等项目建设。积极做好京沪高铁二通道、济莱临连高铁、临沂至滕州高速、临沂至临淄高速、董梁高速临沂段等项目前期工作。开工建设蒙山高架路北延、蒙山大道祊河立交桥改造、武汉路祊河桥、沂蒙南路等重点工程，加快构建快速路、主次干支路级配合理的城市路网系统，缓解中心城区交通拥堵。推进城市精细管理。加强智慧城市建设，实施“互联网+城市管理”行动，构建全域覆盖、全网共享、全程可控的智慧城管新体系。加快建设市政务服务中心等大型停车场和覆盖主城区的智慧停车系统，缓解停车难问题。强化属地和网格管理责任，实现全域保洁。坚持精准服务，推动城管执法向源头化解、前端管理和事前防范转变，着力解决群众反映的热点、痛点、堵点问题。加强县城与主城区联动发展。以棚户区改造和园区建设为重点，优化县城空间和产业结构，科学布局公共服务设施，增强县城对人口、资本、技术、项目的承载能力，在全市逐步形成“龙头引领、众星拱月”组团式城市群。年内棚户区改造新开工2.1万套，改造老旧小区94个、121万平方米；完成农业转移人口市民化20万人左右。

（四）大力实施乡村振兴战略，加快实现强农富民新突破。坚持从城乡双向、农业内外、供需两侧共同发力，全面推进“五个振兴”，打造具有沂蒙特色、现代气派的“富春山居图”。年内市级财政投向乡村振兴资金10亿元，增长39.5%。一要以田园综合体为抓手推动产业振兴。继续抓好30个重点田园综合体，发展农业“新六产”，建设一批集循环农业、创意农业、农事体验于一体的产业融合“样板园”。调整优化农业内部结构，重点发展粮油果菜茶畜等十大特色优质产业，积极推广良种良法良机配套，建设现代农业示范区，进一步打响“生态沂蒙山、优质农产品”品牌。争取新创省级农业标准化生产基地20个、省级整建制农产品质量安全县2个，确保主要农产品有效供给。二要以“两新培育”为重点推动人才振兴。培育新型职业农民，实施鼓励人才留乡、能人回乡、大学生返乡创业三年行动计划，推进浙大临沂现代农业研究院建设，建立农业技术联盟，培训新型职业农民2万人以上。培育新型农业经营主体，推广供销社为民服务中心和金丰公社模式，争取市级以上农业产业化重点龙头企业达到800家，新增家庭农场、农民合作社1200家和1000家。三要以乡风文明为突破推动文化振兴。侯卫东官场笔记实施乡风文明提标行动，力争80%以上村镇达到县级以上文明村镇标准。实施乡村文化兴盛行动，建设一批村史馆、民俗馆、乡村博物馆，培养一批乡土文化能人、非物质文化遗产项目传承人，使乡村成为有历史记忆、地域特色的文化源泉。四要以美丽乡村建设为载体推动生态振兴。深化农村人居环境整治，建设15个市级美丽乡村重点片区、100个市级示范村，带动提升1000个村庄，力争涉农街道基本完成农村无害化卫生厕所改造，美丽乡村覆盖率达到20%。推广土壤改良和病虫害生物防治技术，单位耕地化肥、农药使用量分别下降2.3%和1.25%。实施灾后重点防洪减灾和水利补短板工程，建立“从源头到龙头”的安全供水体系，用两年时间让所有村通上自来水。开展新一轮农村电网升级改造，实施绿色能源示范村镇等重大工程，推动供气设施向农村延伸。抓好“四好农村路”三年集中攻坚，每个中心村（社区）及40%的行政村至少拥有一条宽6米以上的公路，每个县区至少打造2条精品示范美丽公路。五要以治理有效为目标推动组织振兴。加强基层组织建设，深化村民自治。实施经济薄弱村三年“清零攻坚行动”，力争年底前80%以上的村集体经济收入超过5万元。抓好乡村综合治理网络建设，完善基层公共法律服务体系和人民调解组织，落实“一村一警务助理一法律顾问”制度，预防和化解社会矛盾。六要以制度创新为保障推动乡村振兴顺利实施。深化农村集体产权制度改革，加强农村产权流转交易体系建设，争创国家金融服务乡村振兴改革创新试验区，推动更多生产要素投向农村。积极推进《饮用水水源地保护条例》《红色文化保护与传承条例》《美丽乡村条例》出台，不断完善有利于“三农”发展的制度体系，为乡村振兴提供有力有效的法治保障。

各位代表，推动乡村振兴，是全面建成小康社会的重大战略任务。我们一定聚焦“三农”、支持“三农”、服务“三农”，尽快让老区农业强起来、农民富起来、农村美起来！

（五）深化重点领域改革，加快实现发展活力新突破。以刀刃向内的勇气和决心，冲破惯性思维束缚，打破传统路径依赖，把革新除弊作为营造一流营商环境的突破口。围绕事关全局和长远的重大战略部署，突出重点，抓住关键，加强制度创新，努力把改革的步子迈得更大。深化放管服改革。以市场主体满意为导向，根据第三方评估结果改进工作措施。按照全省领先、全国一流标准，优化我市营商环境，为市场主体发展提供更多阳光雨露。紧盯“3124”目标，优化审批服务，全面落实“一次办好”改革任务。以政务服务“无差别受理”为抓手，推进能网上办理的“不见面审批”。当好企业和群众的“店小二”，个性化事项“全程代办”。强化“双随机、一公开”监管，推动联合执法，构建公平公正、规范有效的综合监管体系。按要求完成市县机构改革，厘清权责、理顺关系。加大简政放权力度，落实扩权强县改革政策，推进“市县同权”，促进县域经济加快发展。深化投融资体制改革。市场化运作政府基金，放大政府投资引导效应。推动国有企业管理转型、经营转型，加快实现资产规模化、证券化和运营市场化，增强投融资能力，更好地承担公益性事业建设运营任务。支持金融机构拓展业务，鼓励直接融资，规范民间借贷，创新金融产品，进一步完善金融服务体系。组建市级融资担保集团，设立应急转贷基金，督促银行机构建立无还本续贷企业名单，鼓励金融机构发行小微企业信贷资产支持证券，着力解决企业融资难、融资贵问题。推进股权投资，发展混合所有制经济，激发社会投资潜力。毫不动摇鼓励、支持非公有制经济发展，扎实开展“送政策上门、抓政策落实”活动，真心实意帮助解决实际困难，着力构建“亲”“清”新型政商关系，坚决当好民营企业的贴心人和民营经济发展的促进派！深化生产要素市场化配置改革。在全市域推动生产要素向优势产业、优质企业、高效园区流动，提高资源利用效益。全面建立用地3亩以上企业亩产效益评价机制，按照亩均产出对企业评级定档，根据评价结果实施分类监管和差异化政策，扶持优质企业发展壮大，推动传统企业转型，倒逼落后企业市场出清。巩固提升土地二级市场试点成果，加大标准厂房建设力度，探索“标准地”出让和工业用地弹性年期出让，改革“零土地”技术改造项目审批方式，推动土地集约节约利用。健全生态环境质量改善与污染物排放总量挂钩财政政策，建立能耗、产能交易平台。优化区域发展格局，支持蒙阴县探索绿色发展新路径，打造沂蒙生态文明建设高地；推动全市高新区、经开区和产业园区创新发展、集约发展、特色发展，打造高质量发展高地。深化社会保障制度改革。健全居民医疗保险筹资机制，提高医保基金统筹层次，全面推行药品集中带量采购和按病种付费为主的复合型支付方式，基本实现贫困人口基本医保、大病保险和医疗救助全覆盖。完善社会救助体系，落实好计划生育特殊困难家庭特别扶助制度，探索建立低保、特困人员等困难群众基本生活现金救助、实物救助和救助服务相结合的社会救助方式。加强医疗、医保、医药“三医联动”，争创国家中医药综合改革试验区。抓好退役军人和其他优抚对象信息采集，增强优抚针对性。拓展惠民殡葬改革成果，加大公益性公墓建设力度，推进节地生态安葬。深化财税体制改革。规范市以下财政收入划分办法，明确市与县财政事权和支出责任划分。完善全口径预算管理体系，实行“四本预算”和政府债务收支计划一体化编制，进一步健全“花钱必问效、无效必问责”的预算绩效管理机制。深化开发区改革。突出经济管理职能，优化产业功能布局，探索实行“管委会+公司”模式，促进开发区转型升级、率先发展。

（六）大力保障改善民生，加快实现群众福祉新突破。坚定不移贯彻以人民为中心的发展思想，把让老区人民过上幸福美好生活作为最大追求。坚持民之所望、政之所向，积极而为、量力而行，多为群众办实事、解难事、做好事。重点建设“四个家园”：一是建设温暖家园。努力扩大就业。加强创业大学建设，抓好职业技能培训，帮助高校毕业生、农民工、退役军人、去产能分流人员、困难群体等就业创业，新增城镇就业8万人；抓好农民工工资发放工作，坚决维护劳动者合法权益。优先发展教育。启动全国义务教育优质均衡县（区）创建，开展普通高中扩容提质行动，实施第二期特殊教育提升计划，新建改扩建幼儿园100处、中小学100所、普通高中10所、特殊教育学校5所。推动高等教育融合发展，加快临沂农业科技职业学院、理工职业学院和临沂职业学院新校区建设，提高临沂大学办学内涵，大力引进国内外高校在临沂设立分支机构。积极发展卫生健康事业。建设区域医疗中心，提高村卫生室标准化水平，推进分级诊疗，方便群众就医；加强疾病预防控制，抓好疫苗流通和预防接种管理，全面提高出生人口素质和妇幼健康服务水平。开展“脱贫攻坚巩固提升年”活动。坚持政策不变、投入不降、力度不减、队伍不撤，巩固提升脱贫效果。建立“济临扶贫专项基金”，完善保障性扶贫措施，研究低收入群体认定帮扶办法，筑牢稳定脱贫保障网，决不让贫困户脱贫再返贫。扶助特殊群体。健全关爱老年人、残疾人和农村留守儿童服务体系，做好流动家庭、留守家庭、空巢家庭、单人家庭专项救助，分类提高低保、特困人员和残疾人补贴标准，提高孤儿和困境儿童基本生活费补助标准，打造“善爱沂蒙”品牌。推进“住有所居”。坚持“房住不炒”定位，健全多主体供给、多渠道保障、租购并举的住房制度，增加租赁住房有效供应。二是建设生态家园。不折不扣抓好中央环保督察“回头看”和省级环保督察问题整改，深入开展“四减四增”行动，坚决打好九场标志性战役，确保空气环境质量持续改善、水质优良比例不低于60%、危险废物规范化管理抽查合格率90%以上，让群众看到更多的碧水蓝天。积极开展“绿满沂蒙”行动，实施森林生态廊道、荒山绿化美化彩化、退耕还林还果系列工程，新造林8万亩。严格落实“河长制”“湖长制”，扎实开展“清河行动”，争取3~5年再现“小桥流水、鱼翔浅底”的沂蒙好风光。三是建设平安家园。深化分行业集中打非行动，严格落实“一票否决”和生产安全事故刑事调查、停产整顿、联合惩戒制度，保持铁腕治安的高压态势。严厉打击非法金融活动，持续优化金融生态环境，坚决守住不发生区域性金融风险的底线。深入开展“守护舌尖安全”专项整治行动，积极创建山东省食品安全市，确保群众饮食用药安全。加大平安临沂建设和扫黑除恶专项斗争力度，决不让违法犯罪分子有藏身之地。统筹做好地震、气象、人民防空、应急管理等工作，进一步健全公共安全体系。四是建设文明家园。开展“红色基因代代传”主题活动，深入践行社会主义核心价值观，巩固全国文明城市创建成果。突出红色文化旅游特色，实施十大旅游行动，提升全域旅游发展水平。承办好山东省第九届全民健身运动会，举办第十七届书圣文化节、中国沂河体育节等节会活动。新建一批文体设施，县级“三个一”和乡镇（街道）“两个一”工程覆盖率达到60%。加强社会信用体系建设，政府带头讲诚信，全面清理政府部门拖欠企业款项，坚决杜绝“新官不理旧账”。建立企业诚信和个人信用体系，推行信用“红黑名单”制度，完善守信联合激励和失信联合惩戒机制，让诚信者一路畅通、失信者寸步难行。

各位代表，为人民服务是政府的天职。我们一定待群众如亲人，恪守为民之责，提升民生工作“温度”，增加公共服务“底色”，努力让千万老区群众生活得更加殷实、更为幸福、更有尊严！

三、政府自身建设

各位代表，决胜全面建成小康社会，开启高质量发展新征程，对政府工作提出了新的更高要求。各级政府和全体工作人员必须不忘初心、牢记使命，勤勉敬业、奋发有为，努力创造经得起实践、人民和历史检验的业绩。

提升政治站位。大力弘扬沂蒙精神，把对党忠诚融入政府工作人员血脉和灵魂。自觉用习近平新时代中国特色社会主义思想武装头脑、指导实践、推动工作，树牢“四个意识”，坚定“四个自信”，落实“四个服从”，践行“两个维护”，坚决破除形式主义、官僚主义，严肃查处空泛表态、应景造势、敷衍塞责、出工不出力等问题，确保党的路线方针政策在沂蒙大地落地生根、开花结果。

提升法治水平。坚持法无授权不可为、法定职责必须为，确保政府工作始终在法治轨道上开展。健全重大行政决策公众参与、专家论证、风险评估、合法性审查制度，注重发挥法律顾问、律师的作用，做到依法科学民主决策。自觉接受人大法律和工作监督、政协民主监督、纪检监察监督和社会舆论监督，确保做到依法秉公用权。

提升施政本领。坚持一流标准，跳出临沂看临沂，敢于对标先进、突破自我、敢为人先，推动各项工作不断上水平。增强担当意识，主动攻坚克难、勇挑重担，不为不办找理由，多为办好想办法。发扬斗争精神，对违背原则的言行、黑恶势力、违章建设、污染案件、违法生产等不法行为、不正之风敢于亮剑，勇于斗争。树立开放思维，坚决打破固步自封、自娱自乐的守旧思想束缚，以海纳百川的气魄，集聚八方资源。提高专业素养，加强学习和调研，在学思践悟笃行中培养专业知识、专业能力、专业作风，解决好能力不足问题。

提升工作实效。伟大梦想是拼出来、干出来的。各级政府工作人员要不兴伪事兴务实，对标对表抓落实。坚持目标导向、问题导向、结果导向和结果评价相统一，实行重点工作清单制度，明确时间表、路线图、责任书，跟进抓部署、抓督查、抓考核、抓奖惩，确保工作善始善终、善作善成。坚持说实情、办实事、报实数，绝不允许数字造假。健全激励约束机制，营造鼓励担当、容错纠错的工作环境，调动各方积极性，凝聚发展正能量。

提升清廉形象。坚定不移推进政府廉政建设，严管公共资金、公共资源、公共资产、公共工程，从体制机制上铲除滋生腐败的土壤，以制度建设规范权力运行。构建主动发布、政策解读、回应关切的政务公开新格局，真正让权力在阳光下运行。紧盯重大工程、重点领域、关键岗位，强化对权力集中、资金密集、资源富集部门和行业的监督，以执纪问责促进政风转变。坚持无禁区、全覆盖、零容忍，严肃查处侵害群众利益的不正之风和腐败问题。坚决惩治放纵、包庇黑恶势力甚至充当“保护伞”的公职人员，着力打造一支为民务实清廉的人民公仆队伍。

各位代表，使命呼唤担当，奋斗成就未来。让我们紧密团结在以习近平同志为核心的党中央周围，在省委、省政府和市委的坚强领导下，始终保持永不懈怠的精神状态、一往无前的奋斗姿态，顺势而为、乘势而上，开拓进取、拼搏实干，奋力开创新时代大美新临沂建设新局面，以优异成绩向新中国成立70周年献礼！
